# Early Tumor Response and Safety of Atezolizumab Plus Bevacizumab for Patients with Unresectable Hepatocellular Carcinoma in Real-World Practice

**DOI:** 10.3390/cancers13163958

**Published:** 2021-08-05

**Authors:** Yuwa Ando, Tomokazu Kawaoka, Masanari Kosaka, Yuki Shirane, Yusuke Johira, Ryoichi Miura, Serami Murakami, Shigeki Yano, Kei Amioka, Kensuke Naruto, Yumi Kosaka, Shinsuke Uchikawa, Kenichiro Kodama, Hatsue Fujino, Takashi Nakahara, Atsushi Ono, Eisuke Murakami, Masami Yamauchi, Wataru Okamoto, Shoichi Takahashi, Michio Imamura, Kazuaki Chayama, Hiroshi Aikata

**Affiliations:** 1 Department of Gastroenterology and Metabolism, Graduate School of Biomedical and Health Sciences, Hiroshima University, Hiroshima 734-8551, Japan; yuwando@hiroshima-u.ac.jp (Y.A.); kawaokatomo@hiroshima-u.ac.jp (T.K.); m0607k@hiroshima-u.ac.jp (M.K.); yuki0415@hiroshima-u.ac.jp (Y.S.); jyusuke9@hiroshima-u.ac.jp (Y.J.); ryoichim@hiroshima-u.ac.jp (R.M.); serami@hiroshima-u.ac.jp (S.M.); yano0319@hiroshima-u.ac.jp (S.Y.); amioka@hiroshima-u.ac.jp (K.A.); uzumaki1@hiroshima-u.ac.jp (K.N.); eryu-f-airy1225@hiroshima-u.ac.jp (Y.K.); shinuchi@hiroshima-u.ac.jp (S.U.); kodama@hiroshima-u.ac.jp (K.K.); fujino920@hiroshima-u.ac.jp (H.F.); nakahara@hiroshima-u.ac.jp (T.N.); atsushi-o@hiroshima-u.ac.jp (A.O.); emusuke@hiroshima-u.ac.jp (E.M.); myamauchi@hiroshima-u.ac.jp (M.Y.); shoichit@hiroshima-u.ac.jp (S.T.); mimamura@hiroshima-u.ac.jp (M.I.); 2 Department of Clinical Oncology, Hiroshima University Hospital, Hiroshima 734-8551, Japan; wokamoto@hiroshima-u.ac.jp; 3 Collaborative Research Laboratory of Medical Innovation, Hiroshima University, Hiroshima 734-8551, Japan; chayama@mba.ocn.ne.jp; 4 Research Center for Hepatology and Gastroenterology, Hiroshima University, Hiroshima 734-8551, Japan; 5 RIKEN Center for Integrative Medical Sciences, Yokohama 230-0045, Japan

**Keywords:** hepatocellular carcinoma, atezolizumab, bevacizumab, immune checkpoint inhibitor, tumor marker, α-fetoprotein, real-world practice, molecular targeted agents

## Abstract

**Simple Summary:**

Although atezolizumab plus bevacizumab combination therapy was approved in September 2020 as the first immune-combined therapy for hepatocellular carcinoma, the efficacy and safety of this therapy are unclear in real-world practice. This study investigated the early antitumor effects of atezolizumab plus bevacizumab using imaging and tumor markers, and early safety using the frequency of adverse events and the change in hepatic reserve function, especially for patients with a history of previous systemic treatment. The decreased level of serum α-fetoprotein may reflect the early tumor response in atezolizumab plus bevacizumab, and this therapy is safe in all patients, including those in which it was used as a second-line or later treatment.

**Abstract:**

The aim of this study was to investigate the early tumor response and safety of atezolizumab plus bevacizumab for patients with unresectable hepatocellular carcinoma in real-world practice. Forty patients with Child-Pugh class A liver function and eastern cooperative oncology group performance status 0 or 1 were enrolled. The objective response rate (ORR) at six weeks after the start of treatment, changes in α-fetoprotein (AFP) and des-γ-carboxyprothrombin, incidence of adverse events (AEs), and changes in albumin-bilirubin (ALBI) score and serum ammonia level, were evaluated. Among 40 patients, 24 had histories of prior molecular targeted agents (MTAs). The ORR was 22.5% based on mRECIST. Multivariate analysis showed that an AFP ratio <1.0 at three weeks (odds ratio 39.2, 95% confidence interval CI 2.37–649.0, *p* = 0.0103) was the only significant factor for predicting early response. There was no significant difference in the frequency of AEs between patients receiving first-line treatments and others. Fatigue, proteinuria, and ascites were more frequent in patients who experienced prior treatment. No decrease in ALBI score or increase in serum ammonia level was observed. Our study demonstrated that AFP may be useful in assessing early response and that this treatment is safe, including in patients with prior MTA treatments.

## 1. Introduction

Hepatocellular carcinoma (HCC) is one of the leading causes of cancer-related death worldwide [1]. HCC occurs commonly in patients with chronic hepatitis or liver cirrhosis secondary to either hepatitis B virus (HBV), hepatitis C virus (HCV), excessive alcohol intake, non-alcoholic steatohepatitis, or diabetes [2].

The Atezolizumab plus bevacizumab (Atezo + Bev) combination therapy was approved in 2020 as the first immune-combined therapy for HCC. Atezolizumab is a humanized monoclonal antibody programmed to cell death ligand 1 (PD-L1) that blocks the binding of PD-L1 to PD-1 and restores anti-cancer immunity [3]. Bevacizumab targets the vascular endothelial growth factor for angiogenesis and tumor growth [4,5]. In the IMbrave150 trial, Atezo + Bev treatment resulted in maintaining the quality of life of patients and had better survival benefits than sorafenib [6]. As a result, Atezo + Bev is highly anticipated as a useful systemic therapy for HCC, and is also positioned as a first-line treatment [7,8]. 

Currently, six systemic therapies have been approved for the treatment of unresectable HCC: Atezo + Bev, lenvatinib, and sorafenib as first-line treatments; and regorafenib, cabozantinib, and ramucirumab as second-line treatments. Multidrug sequential therapy is available, and it is necessary to switch drugs appropriately. Therefore, it is extremely important to assess the antitumor effect at an early stage of treatment. Although Atezo + Bev is often administered to patients with a history of other systemic therapies in clinical practice, the efficacy and safety for patients receiving Atezo + Bev as a second-line treatment or later are unknown. In the present study, we examined the factors that predicted early tumor response to Atezo + Bev, and investigated the efficacy and safety for patients initiated as first-line, second-line or later treatment.

## 2. Materials and Methods

### 2.1. Patients

The subjects of this retrospective cohort study were 40 patients with unresectable HCC, Child-Pugh class A liver function, and Eastern Cooperative Oncology Group performance status (ECOG PS) 0 or 1. They were treated with Atezo + Bev at our hospital from September 2020 to March 2021, and the therapeutic effect was evaluated by imaging at least once during the observation period. We examined their records and collected their clinical data obtained during treatment. Patients positive for anti-HCV antibodies were considered to have HCC due to HCV, while those positive for HBV surface antigens were judged to have HCC due to HBV. Signs of portal hypertension were defined as having any findings of splenomegaly, portosystemic collaterals, or gastroesophageal varices with endoscopic examination.

### 2.2. Treatment Regimens

Patients received 1200 mg of atezolizumab plus 15 mg of bevacizumab, per kilogram of body weight, intravenously every 3 weeks. Interruptions to treatment and dose modification were permitted for adverse drug reactions and the patient’s general condition. Patients continued the therapy until death or if one of the following criteria was met for the cessation of therapy: progressive disease following treatments, adverse events that required termination of treatment, deterioration of ECOG PS to 4, worsening liver function, or withdrawal of consent.

### 2.3. Assessment of Response to Therapy

Imaging evaluation was performed according to the Response Evaluation Criteria in the Solid Tumors (RECIST) guidelines [9] and modified RECIST (mRECIST) guidelines [10] with computed tomography and magnetic resonance imaging 6 weeks after the start of treatment. Serum α-fetoprotein (AFP) and des-γ-carboxyprothrombin (DCP) levels were measured 3 and 6 weeks after the start of treatment. 

The AFP ratio was calculated using the following equations: AFP ratio at 3 weeks = (AFP value at 3 weeks)/(AFP value before treatment), and AFP ratio at 6 weeks = (AFP value at 6 weeks)/(AFP value before treatment). The DCP ratio was similarly calculated. When the tumor markers remained in the normal range before treatment, at 3 weeks, and 6 weeks, the ratio was calculated as 1.

### 2.4. Safety Assessment

Treatment safety was examined using adverse events (AEs) and changes in albumin-bilirubin (ALBI) score and serum ammonia level. Adverse drug reactions were defined according to the Common Terminology Criteria for Adverse Events (CTCAE) version 5.0. The ALBI scores were determined from laboratory test results for albumin and total bilirubin, where available, using the equation ALBI score = (log_10_ bilirubin [μmol/L] × 0.66) + (albumin [g/L] × −0.085) [11]. Modified ALBI (mALBI) grades were assigned according to the ALBI scores, wherein an ALBI score ≤ −2.60 was grade 1, −2.60 < an ALBI score ≤ −2.27 was grade 2a, −2.27 < an ALBI score ≤ −1.39 was grade 2b, and an ALBI score > −1.39 was grade 3 [12].

### 2.5. Statistical Analysis

The statistical analyses included the chi-square test or Fisher’s exact test, Mann–Whitney U-test, Wilcoxon rank-sum test, logistic regression analysis, Bonferroni’s multiple comparison test. Continuous variables were expressed as means or medians, while categorical variables were expressed as absolute and relative frequencies.

A *p*-value < 0.05 denoted a statistically significant difference. All statistical analyses were carried out using Predictive Analytics Software R version 3.3.2 [13]. 

## 3. Results

### 3.1. Patient Background Characteristics

The clinical characteristics of 40 patients enrolled in the present study at the initiation of Atezo + Bev are shown in Table 1. This group consisted of 16 patients (40%) treated with Atezo + Bev as first-line treatment and 24 patients (60%) with a history of other systemic therapy. There were 30 men and 10 women with a median age of 69 years. Twenty-one (52.5%) had a Child-Pugh score of 5, and 19 (47.5%) had a Child-Pugh score of 6 for hepatic reserve function. On the other hand, there were 23 patients (57.5%) with modified albumin-bilirubin (mALBI) grades 1 or 2a, and 17 patients (42.5%) with an mALBI grade 2b. The median observation period was 120 days. Compared with the second-line or later group, the first-line group displayed better ALBI scores (median was −2.65 in the first-line group and –2.24 in the second-line or later group, *p* = 0.048) and less extrahepatic metastasis (18.8% in the first-line group and 54.2% in the second-line or later group, *p* = 0.047). 

Table 2 summarizes the contents of pretreatment in 24 patients who initiated Atezo + Bev as second-line or later treatment. This group included 16 patients treated as second-line, 2 patients treated as third-line, 4 patients treated as fourth-line and 2 patients treated as fifth-line.

### 3.2. Tumor Responses

Based on the mRECIST guideline, the proportion of patients with a complete response (CR), partial response (PR), stable disease (SD), and progressive disease (PD) at the first evaluation, performed 6 weeks after the start of treatment, was 0.0%, 22.5%, 47.5%, and 27.5%, respectively. The objective response rate (ORR = CR + PR) was 22.5%. In contrast, based on the RECIST guideline, the proportion of patients with CR, PR, SD, and PD was 0.0%, 7.5%, 65.0%, and 27.5%, respectively, and the ORR was 7.5%. There was no statistically significant difference in ORR between the first-line group and the second-line or later group (*p* = 0.12 and *p* = 1.0, respectively) (Table 3)

### 3.3. Changes in Tumor Markers

**Figure 1:**
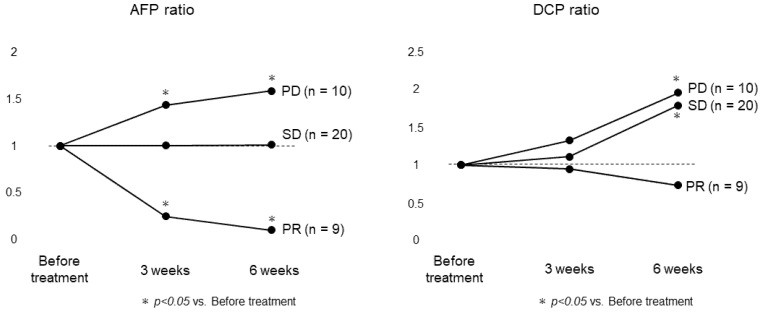
Changes in AFP and DCP ratios for each response based on mRECIST. In 9 patients with partial response, the AFP ratio at 3 and 6 weeks decreased with a statistically significant difference, and increased with a statistically significant difference in 10 patients with progressive disease. On the other hand, the DCP ratio increased with a statistically significant difference at 6 weeks in patients with stable disease or progressive disease.

Figure 1 shows the changes in the AFP and DCP ratios at 3 weeks and 6 weeks after the initiation of treatment. The AFP ratio at 3 and 6 weeks decreased significantly in nine patients who were judged to be PR by mRECIST guidelines, and increased significantly in 10 patients who were judged to be PD. On the other hand, the DCP ratio at 6 weeks increased significantly in patients with SD and PD.

### 3.4. Correlation between Assessment by Tumor Markers and mRECIST

We examined the factors that predicted the response at first evaluation using the background factors at the initiation of treatment and the AFP and DCP ratios at 3 weeks. Univariate analysis showed a relationship between the response at first evaluation and Child-Pugh score, serum DCP level at the start of treatment and AFP ratio at 3 weeks. Multivariate analysis showed that an AFP ratio < 1.0 at 3 weeks (odds ratio, 21.3; 95% confidence interval, 2.01–225.0; *p* = 0.011) was the only significant and independent predictor of the early antitumor response (Table 4). Twelve patients had an AFP ratio of less than 1 at 3 weeks, and six of them were judged to be PR (ORR = 50%). On the other hand, 27 patients had an AFP ratio of 1 or higher at 3 weeks, and four of them were judged to be PR (ORR = 11.1%).

### 3.5. Adverse Events

**Figure 2:**
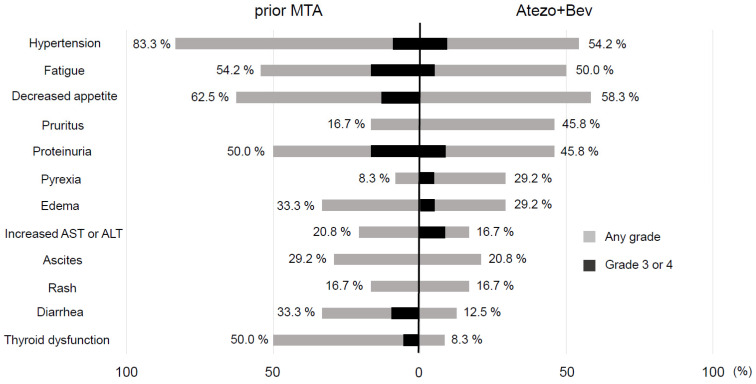
Comparison of adverse events between prior MTA therapy and atezolizumab plus bevacizumab. Adverse events found in >50% included hypertension, fatigue, decreased appetite, proteinuria, and thyroid dysfunction with prior MTA therapy, while hypertension, fatigue decreased appetite were seen with atezolizumab plus bevacizumab.

AEs that occurred during treatment are summarized in Table 5. The most common adverse events were hypertension (24 cases [60.0%]), followed by fatigue (23 cases [57.5%]), decreased appetite (23 cases [57.5%]) and pruritus (20 cases [50.0%]). Regarding the adverse events of CTCAE Grade 3 or higher, hypertension was observed in four cases (10.0%), and proteinuria, increased aspartate aminotransferase (AST) or alanine aminotransferase (ALT), and gastrointestinal bleeding were seen in three cases (7.5%). There was no statistically significant difference in the incidence of any adverse events between the groups treated as first-line or, second-line or later treatment. Among the eight patients with liver dysfunction, three required corticosteroid treatments. Furthermore, two patients were treated with mycophenolate mofetil. In 24 patients previously treated with other MTAs, the frequency of major adverse events was compared between prior MTA therapy and Atezo + Bev (Figure 2). AEs found in ≥50% symptoms such as a decreased appetite, hypertension, fatigue, proteinuria, and thyroid dysfunction with prior MTA treatment, whereas decreased appetite, hypertension, and fatigue were seen with Atezo+Bev. Although the incidence of many AEs with Atezo+Bev was lower than with previous MTA treatment, AST or ALT elevations of CTCAE grade 3 or 4 was more frequent with Atezo + Bev. Furthermore, fatigue, proteinuria, and ascites were more frequent in the patients who had experienced these events with prior MTA treatment (Table 6).

### 3.6. Change in ALBI Score

**Figure 3:**
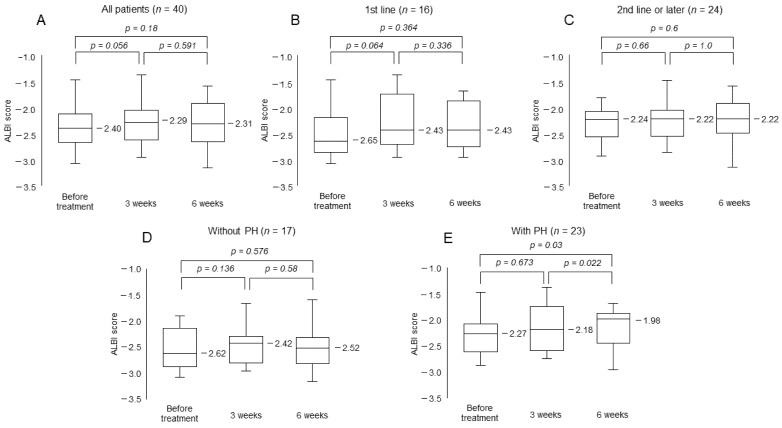
Change in albumin–bilirubin (ALBI) score. (**A**)All patients. (**B**) Patients treated as first-line treatment. (**C**) Patients treated as second-line or later treatment. (**D**) Patients without portal hypertension (PH). (**E**) Patients with PH.

The median ALBI scores of all patients before treatment and at 3 weeks and 6 weeks after treatment were −2.40, −2.29 and −2.31, respectively. No statistically significant difference was observed between any of the time points (Figure 3A). No deterioration in ALBI score was observed in patients initiated as first-line, or second-line and later treatment (Figure 3B,C). On the other hand, the ALBI score significantly worsened in the patients with portal hypertension (Figure 3D,E).

### 3.7. Change in Serum Ammonia Level

**Figure 4:**
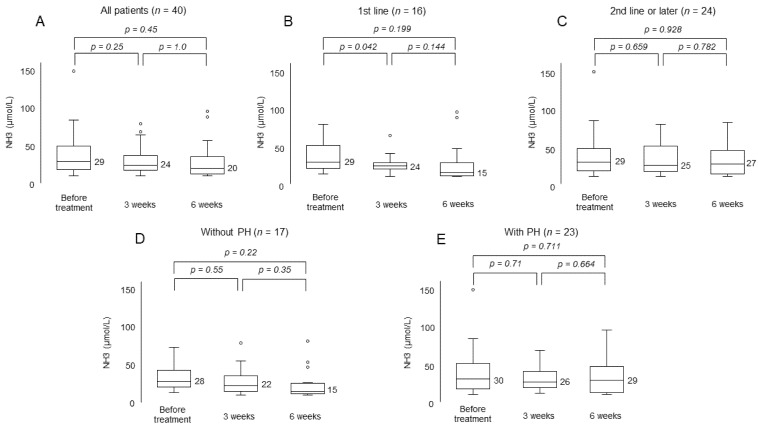
Change in serum ammonia level. (**A**) All patients. (**B**) Patients treated as first-line treatment. (**C**) Patients treated as second-line or later treatment. (**D**) Patients without portal hypertension. (**E**) Patients with portal hypertension. Circles (“°”) mean outliers.

The serum ammonia levels of all patients before treatment and at 3 weeks and 6 weeks after treatment were 29, 24, and 20, respectively. No statistically significant difference was observed between any of the time points (Figure 4A). No increase was observed in the patients treated as either first-line treatment or, second-line or later treatment, nor in the patients with or without portal hypertension (Figure 4B–E).

## 4. Discussion

In the present study, we examined the early tumor response and safety of Atezo + Bev for patients with unresectable HCC in real-world practice. We assessed efficacy using the ORR at first evaluation and changes in tumor markers. Regarding safety, the frequency of AEs in patients treated with Atezo + Bev as first-line treatment and those treated with second-line or later treatment, were compared. In addition, we investigated the association with the incidence of AEs in prior treatment among 24 patients with previous of MTA treatment. To our knowledge, this is the first report to examine the prediction of the initial response of Atezo + Bev and the relationship of AEs between Atezo + Bev and prior MTAs.

Now that a multidrug sequential therapy with six regimens is available for HCC, it is extremely important to assess the early tumor response of the drug. In the IMbrave 150 study, the confirmed ORR was 33.2% based on mRECIST and 27.3% based on RECIST, respectively [6]. Our study showed that the ORR using mRECIST and RECIST was 22.5% and 7.5%, respectively. It has been reported that the median time to respond was 2.8 months in the Atezo + Bev group in a clinical trial [6]. Therefore, further long-term analysis is required for the antitumor effect by best evaluation. On the other hand, some reports on the initial experience of Atezo + Bev in real-world practice have already been reported. According to these reports, the efficacy was similar in patients who were initiated as first-line treatment and in patients who had a history of prior systemic treatment [14,15,16]. However, in our study, the ORR decreased slightly in the second-line or later treatment group, so this finding also needs further investigation. However, it is useful to predict effective cases at an early stage when considering post-treatment. In our study, decreased AFP at in patients at 3 weeks of treatment, was extracted as a factor predicting the early tumor response. Patients with already decreased AFP at 3 weeks had the ORR of 50% in the first evaluation at week 6, whereas patients with increased AFP had the ORR of only 11%. With sorafenib treatment, the relationship between prognosis and the change in AFP has been reported [17,18,19,20]. Furthermore, an early change in AFP levels showed a close relationship to the antitumor effect of lenvatinib [21]. The results of the present study suggest that early changes in AFP levels may also reflect the antitumor effects of Atezo + Bev.

There were no new safety concerns compared to the IMbrave 150 study. In our study, the most common adverse events were hypertension (60.0%), followed by fatigue (57.5%), decreased appetite (57.5%) and pruritus (50.0%). Although, the frequency of AST or ALT elevation was similar, that of pyrexia was relatively high compared to the clinical trial. We used corticosteroids and, if necessary, mycophenolate mofetil for liver damage based on the guidelines [22], but no appropriate management methods have yet been established. Between the first-line group and the second-line or later group, there was no difference in the frequency of any AEs. We also examined the frequency of AEs with Atezo + Bev and with prior MTAs in patients with experience of other MTA treatments. The major side effects of prior MTAs, hypertension, loss of appetite, fatigue, diarrhea, and thyroid dysfunction, were less frequent in Atezo + Bev. On the other hand, pruritus and pyrexia were observed more frequently in Atezo + Bev. In addition, the results demonstrated that fatigue, proteinuria, and ascites were more common in patients who had experienced these events during previous treatments.

In our study, many patients were able to continue treatment without the deterioration of hepatic reserve function. No reduction in ALBI score was observed in patients with or without a history of prior MTAs. It has been reported that hepatic reserve deteriorates early in conventional MTA treatment [23,24]. However, the ALBI score was worse only in patients with portal hypertension. Patients with advanced portal hypertension had decreased hepatic portal vein blood flow and were considered to be in a condition in which hepatic reserve function is likely to decrease. Since it is extremely important to continue treatment while maintaining liver function in the treatment of HCC, appropriate management is essential for these patients. On the other hand, serum ammonia levels did not worsen with or without a history of systemic treatment and portal hypertension. Ohya et al. have reported the early deterioration of serum ammonoia levels in patients treated with lenvatinib [25]. Hyperammonemia is a cause of hepatic encephalopathy, which is a major impediment to the continuation of treatment; however, none of the patients were presented with hepatic encephalopathy in this study. 

The present study has two limitations. Firstly, this study was retrospective with a very small sample size. Secondly, the observation period was very short. Thus, a more definitive conclusion requires a longer observation period and more cases. Nevertheless, we have shown the usefulness of AFP to predict early tumor response and the association of AEs in patients with a history of MTA treatments. We believe that this information will be very useful for the management of Atezo + Bev in real-world practice.

## 5. Conclusion

The AFP may be useful for estimating the early antitumor effect of Atezo + Bev, and Atezo + Bev is a safe treatment for patients with or without prior MTA treatments in real-world practice. 

## Figures and Tables

**Table 1:** Clinical background at initiation of atezolizumab plus bevacizumab combination therapy.

Characteristic	All Patients (*n* = 40)	1st Line Patients (*n* = 16)	2nd Line or Later Patients (*n* = 24)	*p*-Value *
Age (yr), range	69 (47–90)	69 (55–90)	70 (47–87)	0.383
Sex (males/females), *n*	30/10	11/5	19/5	0.482
Etiology (Viral/NBNC), *n*	26/14	11/5	15/9	0.746
Child-Pugh score (5/6), *n*	21/19	11/5	10/14	0.117
Modified ALBI grade (1–2a/2b), *n*	23/17	12/4	11/13	0.104
ALBI score	−2.40 (–3.07–1.47)	–2.65 (–3.07–1.47)	–2.24 (–2.94–1.83)	0.048
Serum ammonia level (μg/dL), range	29 (10–149)	29 (13–79)	29 (10–149)	0.793
Size of main tumor (intrahepatic) (mm), range	40 (0–130)	40 (0–130)	38 (0–130)	0.934
Relative tumor size (<50%/≥50%), *n*	36/4	16/0	20/4	0.278
MVI (absent/present), *n*	28/12	11/5	17/7	1.0
EHM (absent/present), *n*	24/16	13/3	11/13	0.047
BCLC stage (B/C), *n*	18/22	9/7	9/15	0.335
Serum AFP level (ng /mL), range	79.0 (1.8–35,780)	174.1 (2.3–35,780)	79.0 (1.8–34,200)	0.730
Serum DCP level (mAU/mL), range	347 (15–108,710)	292 (19–35,040)	446 (15–108,710)	0.945
Observation period (day), range	120 (42–174)	100 (42–184)	136 (46–188)	0.204

* Fisher or chi-squared test, Mann–Whitney U-test. NBNC, non-B non-C viral hepatitis; ALBI, albumin–bilirubin; MVI, macrovascular invasion; EHM, extrahepatic metastasis; BCLC, Barcelona clinic liver cancer; AFP, alpha-fetoprotein; DCP, des-γ-carboxy prothrombin.

**Table 2:** Pretreatment of patients with a history of systemic therapy.

Line	Treatment	*n*
2nd line	Len	14
	Sor	1
	Investigational products	1
3rd line	Len→Sor	1
	Len→Ram	1
4th line	Len→Sor→Reg	2
	Sor→Reg→Len	2
5th line	Len→Sor→Reg→Ram	1
	Sor→Reg→Len→Ram	1

Len, lenvatinib; Sor, sorafenib; Ram, ramucirumab; and Reg, regorafenib.

**Table 3:** Tumor responses at first evaluation based on mRECIST and RECIST 1.1 guideline.

mRECIST					RECIST 1.1				
% (*n*)	All Patients	1st Line Patients	2nd Line or Later Patients	Difference	% (*n*)	All Patients	1st Line Patients	2nd Line or LaterPatients	Difference
	(*n* = 40)	(*n* = 16)	(*n* = 24)	(*p*-Value *)		(*n* = 40)	(*n* = 16)	(*n* = 24)	(*p*-Value *)
Confirmed ORR	22.5 (9)	37.5 (6)	12.5 (3)	0.12	Confirmed ORR	7.5 (3)	6.2 (1)	8.3 (2)	1
CR	0 (0)	0 (0)	0 (0)		CR	0 (0)	0 (0)	0 (0)	
PR	22.5 (9)	37.5 (6)	12.5 (3)		PR	7.5 (3)	6.2 (1)	8.3 (2)	
SD	50.0 (20)	43.8 (7)	54.2 (13)		SD	67.5 (27)	81.3 (13)	58.4 (14)	
PD	25.0 (10)	12.5 (2)	33.3 (8)		PD	25.0 (10)	12.5 (2)	33.3 (8)	
N.E	2.5 (1)	6.2 (1)	0 (0)		N.E	0 (0)	0 (0)	0 (0)	
DCR	72.5 (29)	81.3 (13)	66.7 (16)	0.477	DCR	75.0 (30)	87.5 (14)	66.7 (16)	0.473

* Fisher or chi-squared test. ORR, objective response rate; CR, complete response; PR, partial response; SD, stable disease; PD, progressive disease; N.E, not be evaluated; DCR, disease control rate.

**Table 4:** Univariate and multivariate analyses of predictive factors for response at first evaluation in atezolizumab plus bevacizumab combination therapy.

Variable	Univariate		Multivariate	
	*p*-Value *	OR	95% CI	*p*-Value **
Age (yr) (<70 vs. ≥70)	1.0			
Sex (male vs. female)	1.0			
Etiology (Viral vs. NBNC)	0.694			
ECOG performance status (0 vs. 1)	1.0			
Child-Pugh score (5 vs. 6)	0.021			
Modified ALBI grade (1 or 2a vs. 2b)	0.256			
Relative tumor volume (<50% vs. ≥50%)	0.570			
MVI (absent vs present)	0.697			
EHM (absent vs present)	0.272			
Serum AFP level (<400 vs. ≥400)	0.690			
Serum DCP level (<340 vs. ≥340)	0.060			
History of systemic therapy (without vs. with)	0.120			
AFP ratio at 3weeks (<1.0 vs. ≥1.0)	0.014	21.3	2.01–225.0	0.011
DCP ratio at 3weeks (<1.0 vs. ≥1.0)	0.705			

* Fisher or chi-squared test, ** Binomial logistic regression analysis. OR, odds ratio; CI, confidence interval; ECOG, eastern cooperative oncology group; NBNC, non-B non-C viral hepatitis; ALBI, albumin-bilirubin; MVI, macrovascular invasion; EHM, extrahepatic metastasis; BCLC, Barcelona clinic liver cancer; AFP, alpha-fetoprotein; DCP, des-γ-carboxy prothrombin.

**Table 5:** Adverse events associated with treatment.

Event% (*n*)	All Patients (*n* = 40)			1st Line Patients (*n* = 16)		2nd Line or Later Patients (*n* = 24)		Difference (*p*-Value *)
	Any Grade	Grade 3 or 4	Time (Days)	Any Grade	Grade 3 or 4	Any Grade	Grade 3 or 4	
Hypertension	60.0 (24)	10.0 (4)	24 (2–73)	68.8 (11)	12.5 (2)	54.2 (13)	8.3 (2)	0.512
Fatigue	57.5 (23)	2.5 (1)	22 (6–155)	68.8 (11)	0	50.0 (12)	4.2 (1)	0.332
Decreased appetite	57.5 (23)	2.5 (1)	21 (3–155)	56.3 (9)	1 (6.3)	58.3 (14)	0	1.0
Pruritus	50.0 (20)	0	21 (6–85)	56.3 (9)	0	45.8 (11)	0	0.748
Proteinuria	37.5 (15)	7.5 (3)	23 (6–101)	25.0 (4)	6.3 (1)	45.8 (11)	8.3 (2)	0.74
Pyrexia	35.0 (14)	2.5 (1)	11 (4–77)	43.8 (7)	0	29.2 (7)	4.2 (1)	0.5
Edema	25.0 (10)	2.5 (1)	24 (16–133)	18.8 (3)	0	29.2 (7)	4.2 (1)	0.711
Increased AST or ALT	20.0 (8)	7.5 (3)	16 (2–41)	25.0 (4)	6.3 (1)	16.7 (4)	8.3 (2)	0.69
Ascites	15.0 (6)	0	15 (6–69)	6.3 (1)	0	20.8 (5)	0	0.373
Rash	15.0 (6)	0	29 (7–71)	12.5 (2)	0	16.7 (4)	0	1.0
Diarrhea	12.5 (5)	0	21 (15–171)	12.5 (2)	0	12.5 (3)	0	1.0
Thyroid dysfunction	10.0 (4)	0	44 (37–174)	12.5 (2)	0	8.3 (2)	0	1.0
Gastrointestinal bleeding	7.5 (3)	7.5 (3)		6.3 (1)	6.3 (1)	8.3 (2)	8.3 (2)	1.0
Portal vein thrombus	2.5 (1)	0		0	0	4.2 (1)	0	1.0
Stroke	2.5 (1)	0		0	0	4.2 (1)	0	1.0

* Fisher or chi-squared test. AST, aspartate aminotransferase; ALT, alanine aminotransferase.

**Table 6:** Relationship of incidence between prior treatment and atezolizumab plus bevacizumab combination therapy.

		Atezo + Bev			
	Hypertension	(+)	(−)	Incidence rate (%)	*p*-value *
Prior MTA	(+)	11	9	55.0	1.0
	(−)	2	2	50.0	
		Atezo + Bev			
	Fatigue	(+)	(−)	Incidence rate (%)	*p*-value *
Prior MTA	(+)	10	3	76.9	0.012
	(−)	2	9	18.2	
		Atezo + Bev			
	Decreased appetite	(+)	(−)	Incidence rate (%)	*p*-value *
Prior MTA	(+)	10	5	66.7	0.403
	(−)	4	5	44.4	
		Atezo + Bev			
	Pruritus	(+)	(−)	Incidence rate (%)	*p*-value *
Prior MTA	(+)	2	2	50.0	1.0
	(−)	9	11	45.0	
		Atezo + Bev			
	Proteinuria	(+)	(−)	Incidence rate (%)	*p*-value *
Prior MTA	(+)	9	3	75.0	0.012
	(−)	2	10	16.7	
		Atezo + Bev			
	Pyrexia	(+)	(−)	Incidence rate (%)	*p*-value *
Prior MTA	(+)	2	0	100	0.076
	(−)	5	17	22.7	
		Atezo + Bev			
	Edema	(+)	(−)	Incidence rate (%)	*p*-value *
Prior MTA	(+)	2	6	25.0	1.0
	(−)	5	11	31.2	
		Atezo + Bev			
	Increased AST/ALT	(+)	(−)	Incidence rate (%)	*p*-value *
Prior MTA	(+)	1	4	20.0	1.0
	(−)	3	16	15.8	
		Atezo + Bev			
	Ascites	(+)	(−)	Incidence rate (%)	*p*-value *
Prior MTA	(+)	4	3	57.1	0.015
	(−)	1	16	5.9	
		Atezo + Bev			
	Rash	(+)	(−)	Incidence rate (%)	*p*-value *
Prior MTA	(+)	0	4	0	1.0
	(−)	4	16	20.0	
		Atezo + Bev			
	Diarrhea	(+)	(−)	Incidence rate (%)	*p*-value *
Prior MTA	(+)	0	8	0	0.526
	(−)	3	13	18.8	
		Atezo + Bev			
	Thyroid dysfunction	(+)	(−)	Incidence rate (%)	*p*-value *
Prior MTA	(+)	2	10	16.7	0.478
	(−)	0	12	0	

* Fisher or chi-squared test. Atezo, Atezolizumab; Bev, bevacizumab; MTA, molecular targeted agents.

## Data Availability

Data are contained within the article.
